# Disease Acceptance and Stress as Factors Explaining Preoperative Anxiety and the Need for Information in Patients Undergoing Operative Minihysteroscopy

**DOI:** 10.3390/jcm14113659

**Published:** 2025-05-23

**Authors:** Karolina Chmaj-Wierzchowska, Aleksandra Jasielska, Katarzyna Wszołek, Agnieszka Lach, Izabela Stankowska-Mazur, Katarzyna Tomczyk, Adrian Mruczyński, Martyna Niegłos, Aleksandra Wilczyńska, Kinga Bednarek, Marcin Wierzchowski, Maciej Wilczak

**Affiliations:** 1 Department of Maternal and Child Health and Minimally Invasive Surgery, Poznan University of Medical Sciences, 60-535 Poznan, Polandlach.agnieszka95@gmail.com (A.L.); ktomczyk@ump.edu.pl (K.T.); adrianmrucz@gmail.com (A.M.); aleksandra.juckiewicz@gamil.com (A.W.); kinga.bednarek@ump.edu.pl (K.B.); mwil@ump.edu.pl (M.W.); 2 Faculty of Psychology and Cognitive Science, Adam Mickiewicz University, 60-568 Poznan, Poland; izabela.stankowska@amu.edu.pl; 3 Chair and Department of Chemical Technology of Drugs, Poznan University of Medical Sciences, 60-806 Poznan, Poland; mwierzch@ump.edu.pl

**Keywords:** minihysteroscopy, Acceptance of Illness Scale, Anxiety Scale, Need-for-Information Scale

## Abstract

**Background/Objectives:** The purpose of this study is to evaluate the role of disease acceptance and stress intensity in explaining anxiety levels and the need for information among patients undergoing a minihysteroscopy procedure under local anesthesia, in the period preceding operative hysteroscopy. **Methods:** The study included 116 patients who were admitted to the Center for Hysteroscopy under Local Anesthesia at the Heliodor Święcicki Gynecological and Obstetrical Clinical Hospital of Karol Marcinkowski Medical University in Poznań, Poland, from December 2024 to January 2025, for operative hysteroscopy using the GUBBINI Mini Hystero-Resectoscope under local anesthesia (paracervical block with lignocaine). **Results:** A low level of preoperative anxiety (χ^2^ = 19.9; *p* < 0.001) and a moderate need for information about the procedure (χ^2^ = 31.8; *p* < 0.001) were statistically significant among the majority of patients (*n* = 82; 71% vs. *n* = 67; 58%) in the study group before undergoing minihysteroscopy under local anesthesia. **Conclusions:** Stress and anxiety are inherent aspects of surgical intervention and hospitalization. Therefore, it is reasonable to develop preoperative support standards to help reduce stress levels, which, in turn, can lead to better adaptation to surgical intervention.

## 1. Introduction

Hysteroscopic procedures are minimally invasive, with a high safety index, and widely recognized as “the gold standard” for treating pathology in women of all ages [1,2]. The minimally invasive procedure allows for shorter hospitalization and recovery times, thus reducing treatment costs [3,4,5,6].

However, minihysteroscopy often induces significant anxiety, increasing the likelihood of procedural intolerance. Anxiety may reduce the quality of life and the level of satisfaction with the procedure, and may be related to the perception of pain. High anxiety levels, particularly those related to pain rather than the procedure itself, are strong predictors of a patient’s preference for general anesthesia in future interventions [7,8,9]. Various factors can influence the severity of pain (operator experience, procedure duration, reproductive organ abnormalities, and the patient’s psychological profile, including emotional stress, limited emotional awareness, and difficulties in emotional expression and processing) [10,11,12]. Patients’ anxiety related to the treatment process and hospitalization in medical facilities is well known and documented [13,14].

The use of two simple scales—The Amsterdam Preoperative Anxiety and Information Scale (APAIS) and the Visual Analog Scale (VAS)—helps determine appropriate preoperative treatment and postoperative care [15,16,17,18,19].

This study aims to determine the role of disease acceptance and stress intensity in shaping anxiety levels and the need for information among patients undergoing minihysteroscopy under local anesthesia (paracervical block with lignocaine), in the period preceding operative hysteroscopy. It explores how patients’ emotional and psychological responses, including acceptance of their health condition and stress levels, influence their expectations regarding the procedure, their perception of the surgery, and their overall sense of security.

## 2. Materials and Methods

The study included 116 patients who were admitted to the Center for Hysteroscopy under Local Anesthesia at the Heliodor Święcicki Gynecological and Obstetrical Clinical Hospital of Karol Marcinkowski Medical University in Poznań, Poland, from December 2024 to January 2025, for operative hysteroscopy using the GUBBINI Mini Hystero-Resectoscope (Tontarra Medizintechnik, Tuttlingen, Germany), under local anesthesia (paracervical block with lignocaine).

The criteria for qualifying for the procedure included the presence of abnormal uterine bleeding, changes in the uterine cavity such as endometrial polyps or submucosal myomas, endometrial hyperplasia, and endometrial hypertrophy. Patients with no history of sensitivity to lignocaine or ketoprofen who were in phase I of the menstrual cycle or postmenopausal were eligible for inclusion. Exclusion criteria included heavy genital tract bleeding, vaginal and/or cervical inflammation, operative vaginal deliveries (forceps, vacuum), and pregnancy.

A comprehensive medical history was collected, including details on age, weight, height, past surgeries, drug allergies, number and types of previous deliveries, number of miscarriages, history of cervical and endometrial procedures, and overall health status. After undergoing a gynecological examination and transvaginal ultrasound, patients provided informed consent for the hysteroscopic procedure.

An original survey questionnaire served as the research tool. In addition to questions about education, place of residence, marital status, professional status, and symptoms of depression, the following scales were also included: the Acceptance of Illness Scale (AIS), the APAIS, and the VAS. The Acceptance of Illness Scale (AIS) is used to assess the degree to which individuals accept their illness. It consists of eight statements describing the negative consequences of poor health and can be applied to any disease. A higher acceptance of illness is associated with better psychological adjustment and reduced distress. Responses are ranked on a scale from 1 “strongly agree” to 5 “strongly disagree”, with total scores ranging from 8 to 40. Scores between 8 and 18 indicate low acceptance, scores from 19 to 29 indicate moderate acceptance, and scores from 30 to 40 indicate high acceptance of the disease. The APAIS consists of six statements: four measure overall preoperative anxiety (two related to fear of anesthesia and two related to fear of surgery), while the remaining two form a subscale assessing the need for information about anesthesia and surgery. Patients responded to each statement using a 5-point scale, where 1 denoted “not at all” and 5 denoted “tremendously”. The maximum possible score was 20 points for the anxiety subscale and 10 points for the information need subscale. The VAS is a 100 mm-long scale, with one end marked 0 and the other end marked 10. When assessing anxiety or stress levels, patients manually selected the point that best represented their state, where 0 indicated “no anxiety/no stress” and 10 denoted the “highest imaginable anxiety/stress” or “anxiety/stress most severe”. The questionnaires were completed by patients 60 min before the procedure. During the procedure, patients remained conscious and were able to observe it in real time on a monitor.

Description of the procedure: Thirty minutes before the start of minihysteroscopy, each qualified patient received 100 mg of ketoprofen intravenously. Ten minutes before inserting the minihysteroscope into the cervical canal, 10 mL of a 0.1% lignocaine solution was administered pericervically at two points (10 mL at the 4 o’clock position and 10 mL at the 8 o’clock position). A needle with an insertion depth of approximately 2 mm was used (Hystero-Block). The hysteroscopy was performed using only the vaginoscopy approach. A 0.9% NaCl solution was used as the distension medium, with continuous flow and a pressure of 120 mmHg. The procedure was carried out using the GUBBINI Mini Hystero-Resectoscope system.

In our research methodology, we employed several statistical tests to analyze the data. For nonparametric variables, we utilized Spearman’s rank correlation coefficient to measure the strength of association. To compare groups, we applied the Kruskal–Wallis test, in addition to conducting a two-factor ANCOVA to assess the impact of multiple independent variables. For measuring frequencies in independent samples, the chi-squared test of associations was utilized. Furthermore, to examine the influence of an indirect variable, we implemented the Generalized Linear Model (GLM) mediation model. In this model, the dependent variable was preoperative anxiety, the independent variable was the acceptance of illness, and the mediator was the intensity of declarative stress. Statistical significance was set at *p* < 0.05. Data analysis was conducted using Jamovi (version 2.3.21.0).

## 3. Results

The study group consisted of 116 patients admitted to “the Center for Hysteroscopy under Local Anesthesia” for hysteroscopic treatment. The patients ranged in age from 21 to 68 years (*M* = 41.1; SD = 9.88).

### 3.1. Characteristics of the Study Group

The largest group of patients falls within the 31–40 age range (39.7%), followed by those aged 41–50 years (31%). In contrast, patients over 61 years old make up only 5.2%. This indicates that the procedure primarily affects women of reproductive or perimenopausal age, which may influence the level of anxiety related to potential fertility loss or hormonal changes. The majority of patients are married (69%), suggesting potential for emotional support from a partner. Social support may play a crucial role in reducing preoperative anxiety and managing stress. More than half of the patients (55.2%) have higher education, while 26.7% have secondary education. Patients with higher education may have a greater demand for detailed medical information and a higher level of health awareness, which can influence their anxiety and stress levels. Most patients are engaged in mental work (69%), while 22.4% perform physical labor, and 4.3% are retired or not employed. Those working in mentally demanding professions may have greater informational needs and a more analytical approach to medical procedures. Additionally, 37.1% of patients live in villages with <2000 inhabitants, while 32.8% reside in large cities with populations >500,000. These differences in place of residence may affect access to healthcare and information, potentially influencing anxiety levels and the demand for additional explanations. A total of 38.8% of patients have not undergone previous gynecological procedures, which may heighten anxiety when facing their first such experience. Conversely, 29.3% have had surgeries unrelated to gynecology, which could influence their psychological preparedness. Regarding childbirth history, 44% of patients have not had a childbirth, while 30.2% have had two deliveries, and 7% have had three or more. Additionally, 89.7% of patients have not undergone cesarean sections. A lack of experience with invasive surgical procedures may contribute to increased preoperative anxiety levels. The characteristics of the study group are presented in Table 1.

A total of 6% of patients reported depression in combination with other diseases, while 1.7% had depression as a standalone diagnosis. Additionally, 0.9% had both depression and neurosis. Anxiety disorders were present in 3.4% of patients, reflecting the need for targeted psychological support to effectively manage preoperative anxiety. Migraine was reported by 1.7% of patients alone and in combination with depression in 2.6% of cases. The coexistence of migraine and depression may exacerbate stress responses and increase the need for individualized care. Nearly half of the patients (47.4%) reported having other diseases, including heart disease (hypertension, chronic ischemic heart disease), lung disease (asthma), thyroid disease (hypothyroidism, and/or hyperthyroidism), and diabetes. These conditions can complicate the management of stress and anxiety before surgery due to concerns about potential interactions between existing health issues and the surgical procedure. Additionally, 33.6% of patients did not report any comorbid conditions, which may correlate with lower levels of preoperative stress and anxiety compared to those managing multiple health issues. Mental health conditions were identified in a significant portion of the study population, highlighting the importance of addressing psychological factors in preoperative care. The characteristics of the study group, including diseases reported during the medical interview, are presented in Table 2.

### 3.2. Operative Minihysteroscopy

The analysis of clinical diagnoses in the study group revealed that the most common indication for operative minihysteroscopy was endometrial polyps, followed by myomas and hypertrophy or abnormal bleeding. The characteristics of the study group, including clinical diagnosis and the type of procedure performed, are presented in Table 3.

The characteristics of the studied variables, including age, weight, height, obstetric history, lesion size, and procedure duration, are presented in Table 4. The Shapiro–Wilk test results indicate that all variables deviate from a normal distribution.

### 3.3. Psychological Varialbles

The characteristics of the variables, including the AIS, Stress (VAS), APAIS: Anxiety Scale, and Need-for-Information Scale, are presented in Table 5. The Shapiro–Wilk test results indicate that all studied variables deviate from a normal distribution.

The associations between psychological variables are shown in Table 6. The data indicate that as disease acceptance increases, patients report slightly lower levels of stress and preoperative anxiety, while the need for information remains unchanged. Declared stress is strongly positively correlated with preoperative anxiety and weakly positively correlated with the demand for information.

The differences in psychological variables for each disease are shown in Table 7. No statistically significant differences were observed among the diseases studied.

Differences in psychological variables based on prior surgeries are shown in Table 8. Patients who had not undergone surgery reported higher disease acceptance than those who had. The effect size indicates that prior surgery accounts for approximately 5% of the variance, suggesting a small effect. A borderline statistically significant difference was observed for reported stress, with patients who had undergone a cesarean section reporting the highest stress levels compared to other groups.

#### 3.3.1. Psychological Variables—Does Preoperative Anxiety Depend on Previous Surgeries and Current Diagnosis?

To determine whether preoperative anxiety depends on previous surgeries and current diagnosis, two ANCOVA analyses were conducted. The first two-factor ANCOVA, in a 2 (previous surgeries: yes, no) × 3 (diagnosis: polyp, myoma, and hypertrophy/abnormal bleeding) model, was statistically insignificant (F 5, 110) = 0.1248, *p* = 0.987. The second two-factor ANCOVA, in a 4 (prior surgeries: no, other, gynecological, cesarean section) × 3 (diagnosis: polyp, myoma, hypertrophy/abnormal bleeding) model, was also statistically insignificant (F 10, 105) = 0.467, *p* = 0.908. The frequency of low (4–10 points) and high (11–20 points) preoperative anxiety scores was examined. Approximately 71% of patients (*n* = 82) exhibited low anxiety, while 29% (*n* = 34) exhibited high anxiety, χ^2^ (1, *n* = 116) = 19.9, *p* < 0.001. Before hysteroscopy, a statistically significant majority of patients exhibited low anxiety levels.

#### 3.3.2. Psychological Variables—Does the Need for Information in the Preoperative Phase Depend on Previous Surgeries and Current Diagnosis?

To determine whether the need for information in the preoperative phase depends on previous surgeries and current diagnosis, two ANCOVA analyses were conducted. The first two-factor ANCOVA, in a 2 (previous surgeries: yes, no) × 3 (diagnosis: polyp, myoma, hypertrophy/abnormal bleeding) model, was statistically insignificant (F 5, 110) = 1.012, *p* = 0.414. The second two-factor ANCOVA, in a 4 (prior surgeries: no, other, gynecological, cesarean section) × 3 (diagnosis: polyp, myoma, hypertrophy/abnormal bleeding) model, was also statistically insignificant (F 10, 105) = 1.31, *p* = 0.233. The frequency distribution of information needs was analyzed. A low degree (2–4 points) of information need was reported by 18% (*n* = 21) of patients, a medium degree (5–7 points) by 58% (*n* = 67), and a high degree (8–10 points) by 24% (*n* = 28), χ^2^ (1, *n* = 116) = 31.8, *p* < 0.001. Before hysteroscopy, a statistically significant majority of patients manifested a medium degree of information need.

### 3.4. Correlation of Psychological Variables

**Figure 1:**
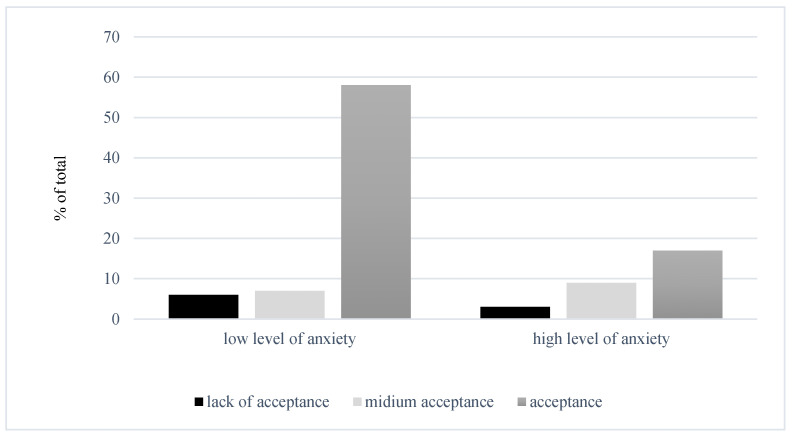
Relationship between the level of preoperative anxiety and the degree of acceptance of the disease.

Analysis of the relationship between preoperative anxiety and disease acceptance was statistically significant, χ^2^ (2, *n* = 116) = 7.93, *p* < 0.001. Cramer’s *V* = 0.261 indicates a weak relationship between these variables. The largest proportion of patients (about 58%) exhibited low anxiety and high disease acceptance. The relationships are illustrated in Figure 1.

**Figure 2:**
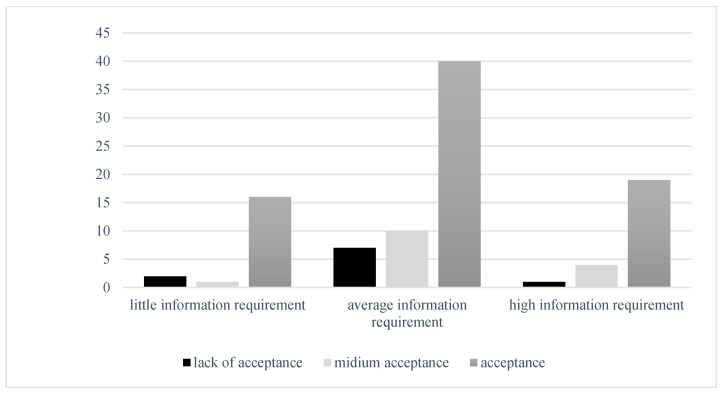
Relationship between the level of preoperative information and the degree of acceptance of the disease.

A statistically significant relationship was not observed between the need for information and disease acceptance, χ^2^ (4, *n* = 116) = 3.95, *p* = 0.413. Regardless of disease acceptance, the need for information remained similar among patients, as shown in Figure 2.

To further explore these relationships, two three-way mediation analyses were conducted. The dependent variable in the first model was preoperative anxiety, while in the second model, it was the demand for information. The independent variable in both models was disease acceptance, with stress intensity as the mediator.

**Figure 3:**
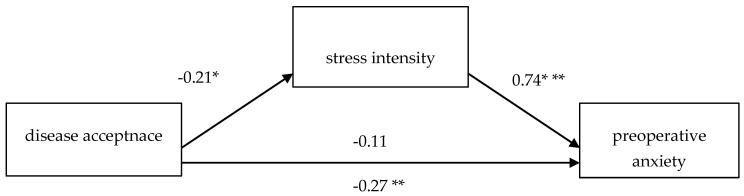
Mediation model for the effect of disease acceptance on preoperative anxiety, with stress intensity as the mediator. Source: own study. * *p* < 0.05; ** *p* <0.01; *** *p* < 0.001.

In the first step of the analysis, a direct relationship between disease acceptance and preoperative anxiety was confirmed. The regression model was a good fit to the data, F(1, 114) = 8.88, *p* < 0.01, indicating that greater disease acceptance was associated with lower preoperative anxiety (β = −0.269, *p* < 0.01). In the second step, the relationship between disease acceptance and stress intensity was also significant—F(1, 114) = 5.27, *p* < 0.05—with β = −0.21, *p* < 0.05. More acceptance was associated with lower perceived stress. In a model incorporating both disease acceptance and perceived stress, disease acceptance was no longer a significant predictor of preoperative anxiety (β = −0.133, *p* = 0.061). However, perceived stress remained strongly associated with preoperative anxiety—F(2, 113) = 84.1, *p* < 0.001. The total mediation model suggests that a direct effect of disease acceptance on reducing preoperative anxiety does not occur, and the only observable pathway of influence is the indirect contribution of stress that accompanies the preoperative period (β = 0.742, *p* < 0.001). A summary of the beta coefficients obtained is presented in Figure 3. The result indicating complete mediation of stress was confirmed by the *Z*-test result = −2.28; *p* < 0.05.

The second mediation model, where information demand was the dependent variable, was found to be insignificant.

## 4. Discussion

The type of surgery and the type anesthesia may also affect the occurrence of preoperative anxiety [20,21,22,23,24]. Maheshwari et al. found that in elective cesarean sections, the prevalence of preoperative anxiety was markedly higher in patients who received general anesthesia than in those who received regional anesthesia (97.18% vs. 51.81%) [25]. Disease acceptance is also associated with preoperative anxiety, with this relationship being fully mediated by stress. This indicates that the level of preoperative anxiety is not solely determined by the degree of disease acceptance but is also influenced by the experience of stress. A low level of preoperative anxiety (χ^2^ = 19.9; *p* < 0.001) and a moderate need for information regarding the procedure (χ^2^ = 31.8; *p* < 0.001) were statistically significant in the majority of patients (*n* = 82; 71% vs. *n* = 67; 58%) in the study group before undergoing minihysteroscopy under local anesthesia. In our previous studies, we did not find any significant differences in anxiety levels and the need for information before hysteroscopy performed under local anesthesia compared with hysteroscopy performed under general anesthesia [14,26]. The anxiety experienced before hysteroscopy is comparable to the anxiety experienced by women waiting for a major surgical procedure under general anesthesia [27]. Preoperative anxiety and stress may also be higher in individuals who have difficulty understanding or identifying emotions, as well as in naming and expressing them (alexithymia), and/or in individuals with low mood or depression [28,29,30,31]. The literature indicates a relationship between disease acceptance and preoperative anxiety. Interested in the earlier results of the study [30], we continued the analysis of only selected variables, in the new group of patients who underwent only surgical hysteroscopy. An analysis of study results by Kozieł et al. [32], involving 112 women diagnosed with breast cancer, demonstrated a negative correlation between anxiety and the level of disease acceptance. This means that the greater the patient’s acceptance of the disease, the lower the intensity of negative reactions and unpleasant emotions associated with it [32]. Similar relationships were observed in the study by Dryhinicz and Rzepa [33], which also confirmed a significant association between anxiety levels and disease acceptance [33]. Moderate-strength negative correlations were observed in both oncological and nononcological patients. However, a key difference emerged between the two groups: in oncological patients, anxiety was more strongly associated with trait anxiety, whereas in nononcological patients, it was linked to state anxiety.

State anxiety is transient and characterized by variability, influenced by threatening factors that emotionally condition the current life situation [34,35]. Trait anxiety, on the other hand, is an acquired and stable behavioral disposition that leads individuals to perceive even objectively nonthreatening situations as dangerous. The anxiety responses in such cases are disproportionate to the actual level of objective danger [36,37,38].

Disease acceptance involves adaptation not only to changes within the body and the patient’s daily life but also in the emotional and cognitive-behavioral domains [39]. The adaptive challenges an individual faces due to illness and the need for surgical treatment represent a significant source of stress. Stress is a typical response to difficult situations, triggering reactions at both psychological and physiological levels of human functioning [40]. These reactions may contribute to heightened anxiety in preoperative situations, as they are categorized as stressful events, representing a natural source of stress.

Anxiety generated by stress is a typical reaction to threats, especially when there are limited options for an effective response [41]. Preoperative anxiety is associated with the experience of losing control, fear of complete dependence on the surgeon and other medical personnel, general anesthesia, the possibility of complications, pain, the risk of death, a cancer diagnosis, loss of fertility, and femininity [42,43,44,45,46]. Moreover, the experience of hospitalization itself, along with the need to function in an unfamiliar physical and social environment, as well as temporary absence from family or professional life, are stress-inducing factors that generate anxiety. Thus, stress accompanies the individual from the moment of hospital admission and the decision to undergo surgery, often leading to doubts about the appropriateness of the planned medical interventions [47]. Preoperative anxiety intensifies the body’s stress response, activating the sympathetic branch of the autonomic nervous system [48].

Anxiety acts as an intermediary in the relationship between personality traits and postoperative outcomes. However, the precise nature of the relationship between personality traits, preoperative anxiety, and postoperative outcomes remains unclear [20]. Based on the above, both stress and anxiety are inherent aspects of surgical intervention and hospitalization. A moderate level of preoperative anxiety is most beneficial for postoperative adaptation [49]. Therefore, it seems appropriate to develop preoperative patient support standards that help reduce stress levels, ultimately leading to better adaptation to surgical intervention. Patients with clinically significant psychological problems should be referred for psychological intervention before the procedure [20].

The analysis of patient characteristics reveals several factors that may influence preoperative anxiety and stress levels in women undergoing operative minihysteroscopy. Social support, particularly from a partner, plays a crucial role in mitigating anxiety, as married patients often benefit from emotional reassurance. Educational background also significantly impacts patients’ informational needs. Those with higher education levels tend to seek more detailed medical explanations, which can help ease their anxiety. Additionally, prior medical experiences—or the absence of such experiences—are important determinants of preoperative stress. Patients who have never undergone surgery may experience heightened anxiety due to unfamiliarity with the process. Lastly, place of residence impacts access to healthcare resources and specialized information. Patients from larger cities often have better access to medical knowledge, which can contribute to lower stress levels. These insights emphasize the importance of personalized support strategies, including tailored medical communication and psychological support, to effectively address the individual needs of patients before operative minihysteroscopy.

The analysis of comorbidities and psychological conditions in patients undergoing operative minihysteroscopy reveals critical factors influencing preoperative anxiety and stress. The high prevalence of comorbid conditions, such as hypertension and chronic diseases, along with mental health disorders like depression and anxiety, highlights the need for a multidisciplinary approach to patient care. Patients with multiple health issues may experience heightened anxiety due to concerns about surgical outcomes and potential complications. Mental health conditions, particularly depression and anxiety, are strong predictors of preoperative stress and should be managed through targeted psychological interventions. Moreover, the absence of comorbidities in some patients may correlate with lower stress levels, although individual psychological resilience can vary.

## 5. Conclusions

In conclusion, implementing comprehensive preoperative assessments, providing personalized psychological support, and delivering detailed medical information can significantly reduce anxiety and improve patient outcomes. Integrating mental health support into surgical care pathways is essential for enhancing the overall experience and well-being of patients undergoing operative minihysteroscopy.

## Figures and Tables

**Table 1:** Characteristics of the study group.

Variable	Category	*n* = 116	(%)
Age	21–30 years	16	13.8%
	31–40 years	46	39.7%
	41–50 years	36	31.0%
	51–60 years	12	10.3%
	>61 years	6	5.2%
Marital status	Single	24	20.7%
	Married	80	69.0%
	Widowed	3	2.6%
	Divorced	9	7.8%
Education	Primary	1	0.9%
	Vocational	7	6.0%
	Secondary	31	26.7%
	Bachelor’s degree	13	11.2%
	Higher	64	55.2%
Occupational status	Retirement	5	4.3%
	Unemploed	5	4.3%
	Manual worker	26	22.4%
	White-collar worker	80	69.0%
Place of residence	Village <2000 citizens	43	37.1%
	City 2000–50,000 citizens	20	17.2%
	City 50,000–200,000 citizens	10	8.6%
	City 200,000–500,000 citizens	5	4.3%
	City > 500,000 citizens	38	32.8%
Previous gynecological surgeries	Cesarean section	12	10.3%
	Surgeries other than gynecological	34	29.3%
	Minor gynecological procedure (abrasio/hysteroscopy)	18	15.5%
	Moderate gynecological procedure	1	0.9%
	Major gynecological surgery	6	5.2%
	No	45	38.8%
Natural childbirth *	0	51	44.0%
	1	22	19.0%
	2	35	30.2%
	3 and more	8	7%

Moderate gynecological procedure (diagnostic laparoscopy, laparoscopic removal of ovarian tumor, fallopian tube, uterine myoma and hysterolaparoscopy); major gynecological surgery (laparotomy/laparoscopic removal of the uterus); * operative vaginal deliveries (forceps, vacuum) were an exclusion criterion for the study.

**Table 2:** Characteristics of the whole study group.

		*n*	(%)
Diseases	Other	55	47.4%
	Other, depression	7	6.0%
	Other, depression, neurosis	1	0.9%
	Other, migraine	3	2.6%
	Other, anxiety disorders	4	3.4%
	Migraine	2	1.7%
	Migraine, depression	3	2.6%
	Depression	2	1.7%
	No	39	33.6%

Other, i.e., comorbidities: heart disease and/or thyroid disease and/or lung disease and/or diabetes.

**Table 3:** Characteristics of the study group, including clinical diagnosis and type of procedure performed.

		*n*	%
Diagnosis	Polyp	90	77.6%
	Hypertrophy/abnormal bleeding	12	10.3%
	Myomas	14	12.1%
Procedure	Operative hysteroscopy; endometrial polyp resection	94	80%
	Operative hysteroscopy; uterine myoma resection	8	6.9%
	Operative hysteroscopy; polypoid endometrial and hyperplasia resection	14	12.1%

**Table 4:** Descriptive statistics: age, weight, height, obstetric interview, lesion size, and procedure time.

Variable	*M*	SD	Min.	Max.	*W*	*p*
Age	41.121	9.885	21	68	0.966	0.005
Weight [kg]	71.940	17.009	48	138	0.903	<0.001
Height [cm]	166.569	6.356	152	178	0.978	0.050
BMI	25.829	5.829	17.4	44.5	0.904	<0.001
Miscarriages	0.147	0.442	0	3	0.370	<0.001
Births	1.198	1.217	0	9	0.748	<0.001
Lesion size during ultrasound [mm]	12.000	5.099	4	25	0.932	<0.001
Procedure time [min]	18.103	6.520	10.0	35.0	0.874	<0.001

**Table 5:** Descriptive statistics: Acceptance of Illness Scale (AIS), Stress (VAS), and APAIS: Anxiety Scale and Need-for-Information Scale.

	*M*	SD	Min.	Max.	*W*	*p*
Acceptance of Illness Scale (AIS)	32.46	8.94	8.00	40.0	0.798	<0.001
Stress (VAS)	4.37	2.20	0	10	0.962	0.002
APAIS: Anxiety Scale	9.34	3.56	4.00	20.0	0.925	<0.001
APAIS: Need-for-Information Scale	6.22	1.85	2.00	10.0	0.961	0.002

**Table 6:** Correlation matrix (rho-Spearman).

	1	2	3	4
1. Acceptance of Illness Scale (AIS)	-			
2. Stress (VAS)	−0.248 *	-		
3. APAIS: Anxiety Scale	−0.315 **	0.746 **		
4. APAIS: Need-for-Information Scale	−0.113	0.251 *	0.367 **	-

* *p* < 0.01, ** *p* < 0.001.

**Table 7:** Psychological factors vs. diagnosis.

	Diagnosis			KW (*df* = 2)	*p*
	Polyp (*n* = 90) *M* (SD)	Myomas (*n* = 12) *M* (SD)	Endometrial Hyperplasia/Abnormal Uterine Bleeding (*n* = 14) *M* (SD)		
Acceptance of Illness Scale (AIS)	32.22 (9.26)	34.08 (7.55)	32.57 (8.27)	0.152	0.927
Stress (VAS)	4.56 (2.13)	3.92 (2.23)	3.57 (2.53)	4.202	0.122
APAIS: Anxiety Scale	14.79 (5.87)	9.25 (2.96)	9.25 (2.96)	1.334	0.513
APAIS: Need-for-Information Scale	6.36 (1.87)	5.67 (1.83)	5.86 (1.75)	1.801	0.406

**Table 8:** Psychological factors vs. previous surgeries.

	Previous Surgeries				KW (*df* = 1)	*p*
	No (*n* = 45) *M* (SD)	Yes (*n* = 71) *M* (SD)				
Acceptance of Illness Scale (AIS)	34.58 (8.08)	31.11 (9.25)			5.83231	0.016 *ε*^2^ *=* 0.05072
Stress (VAS)	4.49 (2.46)	4.30 (2.03)			0.16347	0.686
APAIS: Anxiety Scale	9.31 (3.87)	9.37 (3.37)			0.15930	0.690
APAIS: Need-for-Information Scale	6.20 (1.95)	6.24 (1.80)			0.00119	0.972
		Other (*n* = 35) *M* (SD)	Gynecolo-gical (*n* = 24) *M* (SD)	Cesarean section (*n* = 12) *M* (SD)	(*df* = 3)	
Acceptance of Illness Scale (AIS)	34.58 (8.08)	32.06 (8.38)	30.63 (9.50)	29.33 (11.47)	6.22	0.101
Stress (VAS)	4.49 (2.46)	3.74 (1.93)	4.58 (2.04)	5.33 (1.92)	7.22	0.065
APAIS: Anxiety Scale	9.31 (3.87)	8.77 (3.44)	9.38 (2.73)	11.08 (3.99)	5.14	0.162
APAIS: Need-for-Information Scale	6.20 (1.95)	6.40 (1.91)	5.96 (1.49)	6.33 (2.10)	1.75	0.626

## Data Availability

The data presented in this study are available on request from the corresponding author.
